# The Epstein-Barr virus EBNA1 protein binds to G-quadruplexes in its own mRNA hence controlling its expression and immune evasion of the virus

**DOI:** 10.1093/nar/gkaf586

**Published:** 2025-07-04

**Authors:** Van-Trang Dinh, Nadège Loaëc, Alicia Quillévéré, Marc Keruzoré, Aline Peynet, Ronan Le Sénéchal, Marie-Paule Teulade-Fichou, Laurent Corcos, Rodrigo Prado Martins, Anton Granzhan, Marc Blondel

**Affiliations:** Univ Brest; Inserm UMR1078; Etablissement Français du Sang (EFS) Bretagne; CHRU Brest, Hôpital Morvan, Laboratoire de Génétique Moléculaire, 22 avenue Camille Desmoulins, F-29200 Brest, France; Univ Brest; Inserm UMR1078; Etablissement Français du Sang (EFS) Bretagne; CHRU Brest, Hôpital Morvan, Laboratoire de Génétique Moléculaire, 22 avenue Camille Desmoulins, F-29200 Brest, France; Univ Brest; Inserm UMR1078; Etablissement Français du Sang (EFS) Bretagne; CHRU Brest, Hôpital Morvan, Laboratoire de Génétique Moléculaire, 22 avenue Camille Desmoulins, F-29200 Brest, France; Univ Brest; Inserm UMR1078; Etablissement Français du Sang (EFS) Bretagne; CHRU Brest, Hôpital Morvan, Laboratoire de Génétique Moléculaire, 22 avenue Camille Desmoulins, F-29200 Brest, France; Univ Brest; Inserm UMR1078; Etablissement Français du Sang (EFS) Bretagne; CHRU Brest, Hôpital Morvan, Laboratoire de Génétique Moléculaire, 22 avenue Camille Desmoulins, F-29200 Brest, France; Univ Brest; Inserm UMR1078; Etablissement Français du Sang (EFS) Bretagne; CHRU Brest, Hôpital Morvan, Laboratoire de Génétique Moléculaire, 22 avenue Camille Desmoulins, F-29200 Brest, France; Chemistry and Modelling for Biology of Cancer (CMBC), CNRS UMR9187, Inserm U1196, Institut Curie, Université Paris Saclay, F-91405 Orsay, France; Univ Brest; Inserm UMR1078; Etablissement Français du Sang (EFS) Bretagne; CHRU Brest, Hôpital Morvan, Laboratoire de Génétique Moléculaire, 22 avenue Camille Desmoulins, F-29200 Brest, France; PFIE, INRAE, UE-1227, F-37380 Nouzilly, France; Chemistry and Modelling for Biology of Cancer (CMBC), CNRS UMR9187, Inserm U1196, Institut Curie, Université Paris Saclay, F-91405 Orsay, France; Univ Brest; Inserm UMR1078; Etablissement Français du Sang (EFS) Bretagne; CHRU Brest, Hôpital Morvan, Laboratoire de Génétique Moléculaire, 22 avenue Camille Desmoulins, F-29200 Brest, France

## Abstract

The oncogenic Epstein-Barr virus (EBV) evades the immune system but has an Achilles heel: its genome maintenance protein (GMP) EBNA1, which is essential for viral genome replication, but also highly antigenic. Hence, the virus evolved a mechanism to limit the translation of *EBNA1* mRNA to the minimum level which allows EBNA1 to fulfil its essential function while minimizing production of EBNA1-derived antigenic peptides. This mechanism involves the binding of the arginine-glycine-rich (RGG) motif of nucleolin (NCL), a host protein, to RNA G-quadruplexes (rG4) of the viral *EBNA1* mRNA. This binding is dependent on arginine methylation of NCL RGG. EBNA1 contains two RGG motifs suggesting it could also be involved in this mechanism. Here we show that EBNA1 binds directly to rG4 of its own mRNA and limits its own expression, depending on its RGG motifs and their arginine methylation. Furthermore, EBNA1 and NCL cooperate to bind to rG4 of *EBNA1* mRNA. As the GMP function of EBNA1 has been previously associated to its ability to bind RNA in an rG4-dependent manner, our results suggest the existence of a ternary EBNA1/NCL/*EBNA1* mRNA protein/RNA complex that serves for both EBNA1 GMP function and capacity to auto-limit its expression to evade the immune system.

## Graphical Abstract

**Graphical Abstract:**
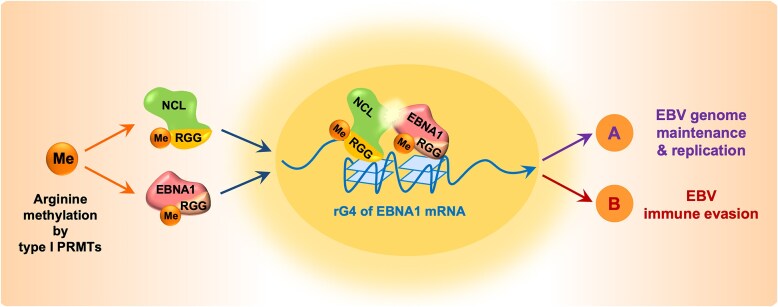

## Introduction

The Epstein-Barr virus (EBV), also known as human herpesvirus 4 (HHV4), was first described as an oncogenic virus in 1964 by Michael A. Epstein and Yvonne Barr [1–4]. EBV is a ubiquitous and highly prevalent virus that infects over 90% of the global human population albeit with some geographical disparities. The majority of EBV infections are not symptomatic, although primary infection in adolescents or young adults can lead to an acute infection known as infectious mononucleosis. After primary infection, EBV persists lifelong in memory B cells and, under specific conditions, can be linked to several human cancers being responsible for at least 1% of cancers worldwide [5], that include 100% of Burkitt lymphomas, 30–50% of Hodgkin lymphomas, 100% of nasopharyngeal carcinoma, 10% of gastric cancers and potentially gliomas [6–8].

In a similar manner to other gamma herpesviruses, EBV is a latent virus capable of evading the host immune system. However, it has an Achilles heel: its Epstein-Barr nuclear antigen 1 (EBNA1) protein, which is the genome maintenance protein (GMP) of EBV [9–11]. Indeed, EBNA1 is essential for EBV genome replication and maintenance and, as such, expressed in all EBV-infected cells, whatever the degree of latency [7]. However, unfortunately for the virus, EBNA1 is also highly antigenic, and EBV-infected individuals contain CD8^+^ T cells raised against EBNA1 [12–14]. Hence, EBV has seemingly developed a mechanism to limit the expression of this protein to the minimal level necessary for its crucial function, thereby also limiting the production of EBNA1-derived antigenic peptides, thus allowing the virus to escape recognition by the immune system [15, 16].

This mechanism of EBNA1 immune evasion directly involves its GAr domain that is composed of repetitions of single alanines separated by one, two or three glycine(s). This GAr domain appears to be important only for the immune evasion of EBNA1, since a form of EBNA1 deleted of its GAr domain (EBNA1ΔGAr) is highly expressed and therefore no longer able to evade the immune system [17, 18], but still able to ensure its essential GMP function. Of note, a natural polymorphism in the length of GAr has been observed and, interestingly, the inhibitory effect of GAr on translation as well as on antigen presentation is GAr-length-dependent: the longer GAr, the less EBNA1 is expressed and the more efficient is its immune evasion [19]. Importantly, the GAr-encoding mRNA sequence is very rich in guanines, organized in multiple putative quadruplex-forming sequences (PQFS), with one of them (termed gEBNA1) repeated up to thirteen times. gEBNA1 has been shown to adopt *in vitro* a G-quadruplex conformation, whose exact structural details are yet unknown [20, 21]. G-quadruplexes (G4) are non-canonical secondary structures that can fold in nucleic acids rich in guanines which, in this structure, interact with each other via Hoogsteen hydrogen bonds. Four guanines form a G-quartet, a square and planar structure. The stacking of several G-quartets (at least two, most often three, or even more), stabilized by monovalent cations, most often K^+^, located in between G-quartets, constitutes a G4 structure. G4 structures have been implicated in various steps of gene regulation such as transcription [22], alternative splicing [23–26] and translation [20, 27–29]. RNA G-quadruplex (rG4) structures that can form in the GAr-encoding mRNA sequence have been proposed to be responsible for the *in cis* inhibition of *EBNA1* mRNA translation by impeding ribosome progression [21].

We have recently developed an original yeast-based model that recapitulates the main features of GAr-based inhibition of *EBNA1* mRNA translation [30–32]. This model allowed to define nucleolin (NCL), a protein widely conserved in eukaryotes, as the first host cell factor critically involved in this mechanism [20]. Indeed, NCL interacts directly with RNA G-quadruplexes (rG4) of *EBNA1* mRNA, and this interaction is necessary for the inhibition of *EBNA1* mRNA translation and antigen presentation, hence allowing immune evasion of EBNA1. We have also shown that several G4 ligands, notably the benchmark compound PhenDC3 [20], and its less toxic analogues such as PhenDH2 or PyDH2 [33], were capable of interfering with the binding of NCL to rG4 of *EBNA1* mRNA. Thus, treatment with these G4 ligands leads to an increase in EBNA1 protein expression and also of its antigenic presentation [20, 33].

Nucleolin is a multifunctional phosphoprotein mostly localized in the nucleolus and the nucleoplasm, but also in the cytoplasm and at the cell membrane [34]. Encoded by the *NCL* gene in humans and the *NSR1* gene in the budding yeast *Saccharomyces cerevisiae*, nucleolin is highly conserved in eukaryotes. Nucleolin is involved in several physiological and pathophysiological processes such as ribosome assembly, chromatin stability, DNA and RNA metabolism, cell proliferation, regulation of apoptosis, as well as in tumorigenesis and viral infection [34–37]. Human nucleolin (NCL) contains four RNA-binding domains called RRM (RNA recognition motifs) and a *C*-terminal region rich in arginine-glycine-glycine, referred to as RGG. Recently, this RGG *C*-terminal motif was shown to be responsible for the capacity of NCL to interact with rG4 of *EBNA1* mRNA, thus playing a crucial role in the inhibition of EBNA1 expression [38]. Moreover, RGG motifs are the main target of arginine methylation, a post-translational modification performed by protein arginine methyltransferases (PRMTs) [39, 40]. The bulk of arginine methylation activity in eukaryotic cells is catalysed by type I PRMTs [41, 42] and we have shown that type I PRMTs, by regulating the interaction of the *C*-terminal RGG motif of NCL with rG4 of *EBNA1* mRNA, control GAr-dependent inhibition of the translation of *EBNA1* mRNA and thereby EBV immune evasion [38]. Therefore, type I PRMTs represent possible intervention points to unveil EBNA1 to the immune system.

Interestingly, EBNA1 protein also contains two RGG motifs, and it has been shown that its genome maintenance function involves its ability to recruit the origin recognition complex (ORC) in an RNA-dependent manner that depends on its two RGG motifs [43]. In addition, EBNA1 protein has been reported to bind to its own RNA [44]. Finally, it has been shown that the two RGG motifs of EBNA1 protein could bind to some rG4 and that this binding is required for the genome maintenance function of EBNA1 [45]. Hence, we sought to determine whether EBNA1 protein, similarly to NCL, could bind in an RGG-dependent manner to rG4 of its own mRNA and, this way, participate to the regulatory mechanism limiting its own expression in order to evade the host immune system. Of note, this model opens the intriguing possibility that the same EBNA1 protein/*EBNA1* mRNA complex may be involved in both the genome maintenance function of EBNA1 and in the mechanism limiting its expression, and thereby its detection by the immune system.

Here we show that EBNA1 protein is able to bind to rG4 of its own mRNA, and that this binding is direct and depends on its two RGG motifs. We then show that EBNA1 protein is able to limit its own expression and that, as for NCL, this ability also depends on its two RGG motifs and on the methylation of arginines of these motifs. Finally, we observed that EBNA1 and NCL proteins, in line with the direct interaction between them, favour the binding of each other to rG4 of *EBNA1* mRNA. Hence, these results suggest the existence of a feedback loop in which, together with the host protein NCL, the EBV-encoded EBNA1 protein participates to the limitation of its own expression. This feedback mechanism ensures that EBNA1 protein is sufficiently expressed to fulfil its genome maintenance function which also involves its ability to bind rG4 [43, 45] and, at the same time, kept below the threshold necessary for recognition by the host immune system.

## Materials and methods

### Yeast strains and culture media

All the yeast *Saccharomyces cerevisiae* strains used in this study are derived from the W303a *WT* K699 strain [46]: *MAT a, leu2-3 112 trp1-1 can1-100 ura3-1 ade2-1 his3-11,15*.

***Y32*** *: MAT a, leu2-3, 113 trp1-1, can 1–100, ura3-1, ade2-1:: his5 ^S.pombe^, his3-11,15, met15::HA-ade2*

***Y32 nsr1* *Δ*** *: MAT a, leu2-3, 113 trp1-1, can 1–100, ura3-1, ade2-1:: his5 ^S.pombe^, his3-11,15, met15::HA-ade2, nsr1::KANMX6*

Yeast strains were used as previously described [38]. The culture media used for yeasts growth were: YPD (1% (w/v) yeast extract, 2% (w/v) peptone, 2% (w/v) glucose)), and DO-TRP (6.7 g*/*L yeast extract without amino acids, 0.74 g of CSM-TRP, 20 g*/*L D-glucose). For solid media, 2% (w/v) agar was added.

### Plasmids constructions

All the plasmids were generated using standard procedures. The T4 DNA ligase was obtained from Promega and the restriction enzymes were purchased from New England Biolabs. The synthesis of the various EBNA1-expressing plasmids was carried out by Genscript. Upon reception, the plasmids were amplified in bacteria. Plasmid extractions were realized using the NucleoBond® Xtra Midi EF kit protocol (Macherey-Nagel). The plasmids were verified by PCR amplification and sequencing.

### Yeast protein extraction

A 4 mL aliquot of exponentially growing yeast cells at 0.6–0.8 OD_600 nm_ was harvested, washed in 1X TE, pelleted and then suspended into 300 mL of lysis buffer (25 mM Tris-HCl pH 6.8, 10% glycerol, 5% β-mercaptoethanol, 5% sodium dodecylsulfate (SDS), 8 M urea, 0.02% bromophenol blue), heated at 95°C and then cooled on ice.

### Cell culture and transfection

Raji cells are from a type III latency EBV-infected Burkitt's lymphoma. H1299 cells are derived from metastatic lymph node from lung carcinoma. These cells were cultured in RPMI-1640 supplemented with 10% foetal bovine serum and 2 mM L-glutamine. Transfections were performed using GeneJuice® transfection reagent (Merck) or jetOPTIMUS® (Polyplus) for H1299 cells, or by electroporation using the Amaxa Cell Line Nucleofector® kit V (Lonza) for Raji cells, in both cases according to the manufacturer protocol.

### Protein extraction from mammalian cells

Whole cells at 80–90% confluency were harvested, washed with 1X PBS and suspended into lysis buffer (20 mM HEPES pH 7.5, 50 mM β-glycerolphosphate, 1 mM EDTA pH 8, 0.5 mM Na_3_VO_4_, 100 mM KCl, 10% glycerol, 1% Triton, anti-proteases cocktail (Roche 11697498001)). These cells suspensions were then mechanically lysed (six series of vortex followed by 10 min incubation on ice) before centrifugation at 16 000 *g* for 20 min at 4°C. Protein concentration was determined using the Bradford assay.

### Western blotting

Equal protein quantities and volumes of all samples were loaded and run on Bolt or NuPAGE^TM^ 10% Bis-Tris Protein gels (Invitrogen), then transferred onto 0.45 μM nitrocellulose membrane (GE Healthcare). Membranes were blocked in 1X PBS, 0.1% Igepal and 3% BSA and incubated with the indicated primary antibodies: mouse anti-GAPDH (Abcam ab125247, 1/5000), rat anti-HA (Roche 11867423001, 1/2000), mouse anti-EBNA1 (Cytobarr OTX1-EBNA1, 1/2000) or rabbit anti-NCL (Abcam ab70493, 1/5000). The membranes were then washed with fresh 1X PBS 0.1% Igepal and incubated with the indicated secondary antibodies conjugated to horseradish peroxidase: rabbit anti-mouse (Dako P0161, 1/3000), swine anti-rabbit (Dako P0217, 1/2000) or goat anti-rat (Millipore AP136P, 1/3000). The membranes were washed afresh and analysed by enhanced chemiluminescence in the following buffer (Tris-base pH 8.5, 12.5 nM coumaric acid, 2.25 nM luminol and 0.15% H_2_O_2_) using a Vilber-Lourmat Photodocumentation Chemistart 5000 imager. All the experiments were repeated independently at least two or three times. Relative proteins levels for each sample were normalized to GAPDH protein levels using the ImageJ software.

### RNA extraction and semi-quantitative RT-PCR

Total Raji cellular RNA were extracted using the RNeasy Plus kit (Qiagen). cDNA synthesis was carried out using 500 ng of RNA and the SuperScript™ IV Reverse Transcriptase (Invitrogen) together with oligo(dT)_20_ primer (Invitrogen). cDNA samples were analysed by semi-quantitative PCR using the MasterMix OneTaq Polymerase (NEB). The relative abundance of target mRNA was normalized using GAPDH as an endogenous control. Quantification of bands density was determined using the ImageJ software. The primers used for PCR were:

- For EBNA1,
- EBNA1-forward: 5′-GGCAGTGGACCTCAAAGAAGAG-3′
- EBNA1-reverse: 5′-CCTGCTCCTGCTCCTGTTCCA-3′
- For GAPDH,
- GAPDH-forward: 5′-GAGTCAACGGATTTGGTCGT-3′
- GAPDH-reverse: 5′-CACAAGCTTCCCGTTCTCAG-3′

All the experiments were performed independently at least three times.

### RNA pulldown

RNA pulldowns experiments were performed as previously described [20, 25, 38, 47]. Briefly, whole cell extracts or recombinant-EBNA1 (Abcam, ab138345) or recombinant-NCL [48] were used for pulldown assays with the following G-quadruplex forming RNA oligonucleotides:

- 2GQ, 5′-GGGGCAGGAGCAGGAGGAGGGGCAGGAG- CAGGAGGA-3′-TEG-Biotin,
- 2GM, 5′-GAGGCAGUAGCAGUAGAAGAGGCAGUAG- CAGUAGAA-3′-TEG-Biotin,
- 1GQ, 5′-GGGGCAGGAGCAGGAGGA-3′-TEG-Biotin,
- 1GM, 5′-GAGGCAGUAGCAGUAGAA-3′-TEG-Biotin

G-quadruplexes were formed by heating the RNA oligonucleotides at 95°C during 5 min and then cooling them down to 4°C at a rate of 2°C per minute in folding buffer (10 mM Tris-HCl pH 7.5, 0.1 mM EDTA) in the presence of 100 mM KCl to promote G4 formation, or in the presence of 100 mM LiCl which does not promote G4 formation. To avoid unspecific binding, high affinity streptavidin-coupled sepharose beads (GE Healthcare, 28985799) were incubated in 1 mL blocking buffer (10 mM Tris-HCl, pH 7.5, 100 mM KCl, 0.1 mM EDTA, 1 mM DTT, 0.01% Triton, 0.1% BSA, 0.02% *S. cerevisiae* tRNAs (Sigma-Aldrich, 10109495001)) for 1 h at 4°C on a rotating wheel. An amount of 5 μg of each folded biotinylated RNA oligonucleotides was incubated with 50 μl of solution containing the streptavidin-coupled sepharose beads for 90 min at 4°C on a rotating wheel. Around 500 μg of cell extracts or 600 ng of recombinant EBNA1 or NCL protein were treated with 200 U/mL of RNase Inhibitor (NEB, M0307S) for 90 min at 4°C on a rotating wheel. These extracts were then incubated with the RNA oligonucleotides bound to the streptavidin beads for 90 min at room temperature. Beads were then washed five times with lysis buffer and lysis buffer with increasing KCl or LiCl concentrations (200–800 mM). Protein still bound to beads after the washes were eluted using 2X loading buffer (2X Laemmli Buffer with 5% β-mercaptoethanol) and analysed by western blotting with antibodies raised against EBNA1, NCL or HA.

### RNA *in situ* hybridization - immunofluorescence (rISH-IF)

H1299 cells were plated on 13-mm-diameter coverslips in 24-well plates and transfected with the indicated constructs. At 24 h post-transfection, cells were fixed with 4% paraformaldehyde for 20 min and then washed with PBS 1X. Cells were then incubated in 70% ethanol for 4–7 h at 4°C. After rehydration in PBS for 30 min, samples were permeabilized with PBS 0.4% Triton X-100, 0.05% CHAPS for 10 min at room temperature and pre-treated with hybridization buffer (10% formamide, 2X SSC, 0.2 mg/mL *E. coli* 522 tRNAs, 0.2 mg/mL sheared salmon sperm DNA and 2 mg/mL BSA) for 30 min at room temperature. Samples were then incubated overnight with 50 ng of an EBNA1-digoxigenin DNA probe (5′-CTTTCCAAACCACCCTCCTTTTTTGCGCCTGCCTCCATCAAAAA-3′-digoxigenin) in a humidified chamber at 37°C. To avoid the formation of secondary structures, the probe was denaturated 5 min at 80°C, then chilled on ice for 5 min, and resuspended in hybridization buffer. After hybridization, samples were serially washed for 20 min with 2X SSC 10% formamide, hybridization buffer (twice), 2X SSC, and PBS. Washes were carried out at room temperature, except with hybridization buffer (37 °C). Samples were next saturated with PBS 3% BSA, 0.1% saponin for 30 min and incubated with a mouse anti-digoxigenin (Sigma, D8156) and a rabbit anti-HA Tag (Invitrogen, PA1985) antibodies for 2 h at room temperature. A goat anti-mouse immunoglobulin G (IgG) secondary antibody conjugated to Alexa Fluor® 594 and goat anti-rabbit immunoglobulin G (IgG) secondary antibody conjugated to Alexa Fluor® 488 (both purchased from Invitrogen) were used to detect immunocomplexes (1 h at 37°C). DAPI was used for nuclear counterstaining under standard conditions and the images were taken using a Zeiss Axio Imager M2.

### Proximity ligation assay (PLA) for protein/protein and protein/mRNA interactions

PLA adapted to monitor protein/RNA interactions was performed as reported previously [20, 49]. Briefly, H1299 cells were fixed as described above for rISH-IF. For experiments using Raji cells, 13-mm-diameter coverslips were coated with poly-L-lysine 0.01% (Sigma) for 30 min and air-dried for 5 min in 24-well plates. Raji cell were next resuspended in PBS, plated on pre-treated coverslips and incubated for 2 h at room temperature. After 20 min of fixation with 4% paraformaldehyde, cells were processed for *in situ* hybridization according to the rISH-IF protocol. Samples were then saturated with 3% BSA in PBS for 30 min and incubated for 2 h at room temperature with a mix of primary antibodies containing the mouse anti-digoxigenin (Sigma, D8156) and the rabbit anti-HA Tag (PA1985, Invitrogen) for H1299 cells or rabbit anti-digoxigenin (D7782, Sigma) and mouse anti-EBNA1 (OT1x-EBNA1, Cytobarr) for Raji cells. Subsequently, PLA was carried out using the Duolink PLA *in situ* kit (Sigma), anti-rabbit plus and anti-mouse minus probes (Sigma) following the manufacturer protocol.

PLA to assess the interaction between NCL and EBNA1 proteins used the same protocol (without the hybridization step), except that the rabbit anti-NCL antibody (ab22758, Abcam) was used together with the mouse anti-EBNA1 antibody (OT1x-EBNA1, Cytobarr) for Raji cells or the mouse anti-HA Tag antibody (a kind gift from Borek Vojtesek, Masaryk Memorial Cancer Institute, Brno, Czech Republic) for H1299 cells. For co-staining of 3HA-EBNA1, samples were incubated overnight with rat anti-HA Tag antibody (ROHAHA, Roche) after PLA amplification step, and a goat anti-rat immunoglobulin G (IgG) secondary antibody conjugated to Alexa Fluor® 488 was used to detect immunocomplexes, or only a goat anti-mouse immunoglobulin G (IgG) secondary antibody conjugated to Alexa Fluor® 488. DAPI was used for nuclear counterstaining and the images were taken using a Zeiss Axio Imager M2. The experiments were repeated twice or more.

### Electrophoretic mobility assay (EMSA)

G-quadruplexes of Cy5.5′-labelled RNA oligonucleotides 2GQ or 2GM (sequences described above in **RNA pulldown** section) were formed by the annealing process described in the **RNA pulldown** section. Then, indicated amounts of recombinant NCL and/or EBNA1 proteins were incubated 30 min at RT with one picomole of 2GQ or 2GM matrix in a 12 μL reaction mixture containing 10 mM Tris-HCl pH 7.5, 0.1 mM EDTA and 100 mM KCl. After incubation, the reaction mixture was subjected to 6% non-denaturing polyacrylamide gel electrophoresis (prepared from an acrylamide/bis-acrylamide 37.5/1 solution) using TBE 0.5X as a running buffer. The gels were then analysed by fluorescence illumination using an Odyssey^TM^ scanner device (LI-COR) and the images were processed using the ImageJ software.

### Co-immunoprecipitation experiments

Extracts from H1299 cells transfected, or not, with both HA-tagged NCL (HA-NCL)- and EBNA1ΔGAr-expressing plasmids were prepared as described in the **Protein extraction from mammalian cells** section. Around 500 μg of the whole cell extracts were incubated with an anti-HA antibody (Invitrogen, PA1985) for 1 h at 4°C. These complexes were then incubated with protein G sepharose beads (Cytiva, 28–9513-79) for 1 h at 4°C on a rotating wheel. Beads were then washed four times with lysis buffer (20 mM HEPES pH 7.5, 50 mM β-glycerolphosphate, 1 mM EDTA pH 8, 0.5 mM Na_3_VO_4_, 100 mM KCl, 10% glycerol, 1% Triton, anti-proteases cocktail (Roche 11697498001)). Protein still bound to beads after the washes were eluted using 2X loading buffer (2X Laemmli Buffer with 5% β-mercaptoethanol) and analysed by western blotting with antibodies raised against HA or EBNA1.

## Results

### EBNA1 protein binds directly and in a G4-dependent manner to rG4 of its own mRNA and this interaction is modulated by G4 ligands

**Figure 1.**
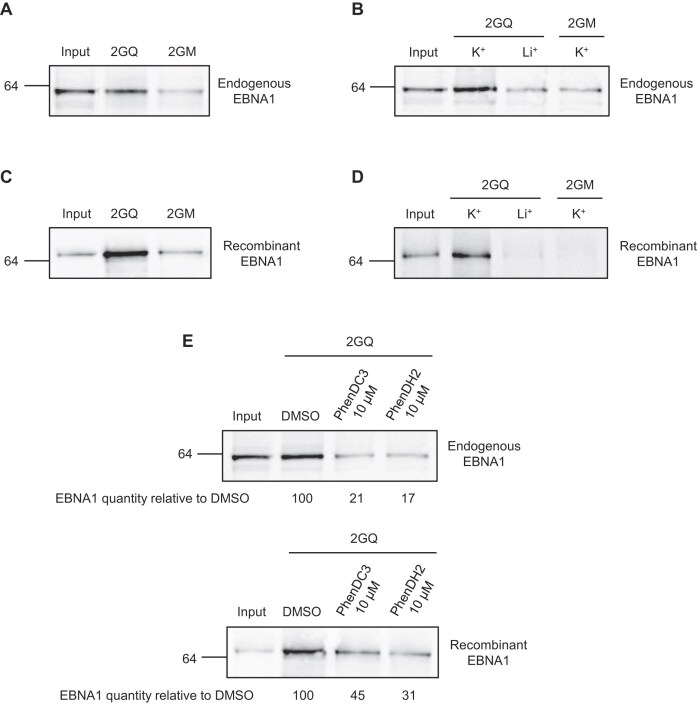
EBNA1 protein binds directly and in a G4-dependent manner to rG4 of its own mRNA and this interaction is modulated by two G4 ligands. (**A**) RNA pulldown using rG4-forming RNA oligonucleotides that are biotinylated and bound to streptavidin-coupled sepharose beads. Extracts from Raji cells (EBV-infected Burkitt lymphoma cells) were loaded on the following matrices: 2GQ (containing two rG4 of *EBNA1* mRNA) or 2GM (same sequence except that four guanines critical for G4 formation were replaced by adenine or uracil). The sequence of these RNA oligonucleotides is indicated in ‘Materials and methods’. The protein still bound after an 800 mM KCl wash were eluted and analysed by SDS-PAGE and western blot using an antibody directed against EBNA1. Blot represents *n* ≥ 3. (**B**) Same experiment as in (**A**), except that RNA matrices were folded in the presence of 100 mM KCl (that favours G4 formation) or, as a control, of 100 mM LiCl (that does not favour G4 formation). Blot represents *n* ≥ 3. (**C**) Same experiment as in (**A**), except that a recombinant polyhistidine-tagged EBNA1 protein was used instead of extracts from Raji cells. Blot represents *n* ≥ 3. (**D**) Same experiment as in (**B**), except that a recombinant polyhistidine-tagged EBNA1 protein was used instead of extracts from Raji cells. Blot represents *n* ≥ 3. (**E**) Same experiment as in (**A**) and (**C**) respectively, in the presence of 10 μM PhenDC3 or 10 μM PhenDH2.

As stated above, EBNA1 protein contains a central glycine-alanine repeat (GAr) which is crucial for its ability to evade the immune system. The GAr-encoding sequence of *EBNA1* mRNA is guanine-rich and is composed of tandem repeats of a multiple and closely similar PQFS [20, 21]. Hence, depending on the length of GAr, which is polymorphic, GAr-encoding RNA sequence may present a cluster of multiple rG4 units. The host cell protein NCL has been involved in GAr-based inhibition of *EBNA1* mRNA translation, hence limiting at the same time the expression of both EBNA1 protein and EBNA1-derived antigenic peptides, thereby allowing immune evasion of EBNA1 and, consequently, of EBV [20]. This role of NCL in immune evasion of EBV critically depends on its ability to bind to the rG4 of the *EBNA1* mRNA through its *C*-terminal arginine-glycine-rich (RGG) motif [38]. As EBNA1 protein itself contains two RGG motifs, one (RGG1) immediately upstream of GAr and the other (RGG2) immediately after GAr, we wondered if EBNA1 protein could also bind to rG4 of its own mRNA. To test this hypothesis, we performed RNA pulldown as previously described [20, 47]. Briefly, this assay is based on the use of biotin-conjugated RNA oligonucleotides (2GQ) containing two copies of gEBNA1, i.e. the most abundant G4-forming sequence of *EBNA1* mRNA. Of note, this dimeric motif *per se* is encountered three times in the GAr-encoding sequence of *EBNA1* mRNA. As a negative control, we used biotin-conjugated oligonucleotides containing the same sequence, except that four guanines critical for rG4 assembly were replaced by adenine or uracil (2GM). As these RNA oligonucleotides are biotin-conjugated, they can be precipitated using magnetic Sepharose beads coupled to streptavidin, thereby allowing affinity precipitation and identification by western blotting of proteins that bind to these sequences. Using extracts from Raji cells, which are B-cells from a type III latency EBV-infected Burkitt's lymphoma and, as such, express EBNA1, at the physiological concentration of KCl (100 mM) which is required for efficient assembly of G4, we observed an efficient binding of endogenous EBNA1 on 2GQ matrix (Fig. 1A). This binding was significantly reduced when using 2GM matrix which is not able to form rG4, readily suggesting that endogenous EBNA1 protein binding on the 2GQ matrix is rG4-dependent. To confirm this result, we repeated the same experiment in the presence of either K^+^ (100 mM KCl), which allows efficient formation of G4, or Li^+^ (100 mM LiCl), which does not. As shown in Fig. 1B, the binding of endogenous EBNA1 on 2GQ matrix was efficient only in the condition where G4 formation is favoured (K^+^) whereas only a residual binding, similar to the one observed for the control 2GM matrix with K^+^, was observed in the condition where G4 formation is not favoured (Li^+^). Altogether these results indicate that EBNA1 protein binds to rG4 structures present in its own mRNA.

The binding of EBNA1 protein to the rG4 of its own mRNA may be indirect and involve other protein(s), for example NCL. To determine if the binding of EBNA1 protein is direct or indirect, we repeated the same RNA pulldown experiments using purified recombinant EBNA1 protein produced in bacteria (recombinant EBNA1). We obtained the same result as when using extracts from Raji cells: recombinant EBNA1 efficiently binds to 2GQ matrix in the presence of K^+^, whereas only a residual binding was observed when using 2GM matrix in the presence of K^+^ (Fig. 1C), or when using 2GQ matrix in the presence of Li^+^ (Fig. 1D). This result indicates that EBNA1 protein binding on the rG4 of its own mRNA is direct.

Next, we tested the effect of PhenDC3 and PhenDH2, two G4-ligands whose ability to interfere with the interaction between NCL and rG4 of EBNA1 was recently described [33], on the interaction between EBNA1 protein and the rG4 of its own mRNA. For this purpose, we conducted similar RNA pulldown experiments as above, but in the presence, or absence, of 10 μM of PhenDC3 or PhenDH2. As shown in Fig. 1E, similarly to their reported effect on NCL/rG4 interaction, both G4 ligands interfere with the ability of EBNA1 protein (endogenously expressed, upper panel or recombinant, lower panel) to interact with rG4 of its own mRNA, confirming that the binding of EBNA1 on its own mRNA is rG4-dependent.

Taken together, all these results indicate that EBNA1 protein directly binds to rG4 of its own mRNA and suggest that this interaction is druggable *via* G4 ligands.

### EBNA1 protein interaction with rG4 of its own mRNA mostly takes place in the nucleus

**Figure 2.**
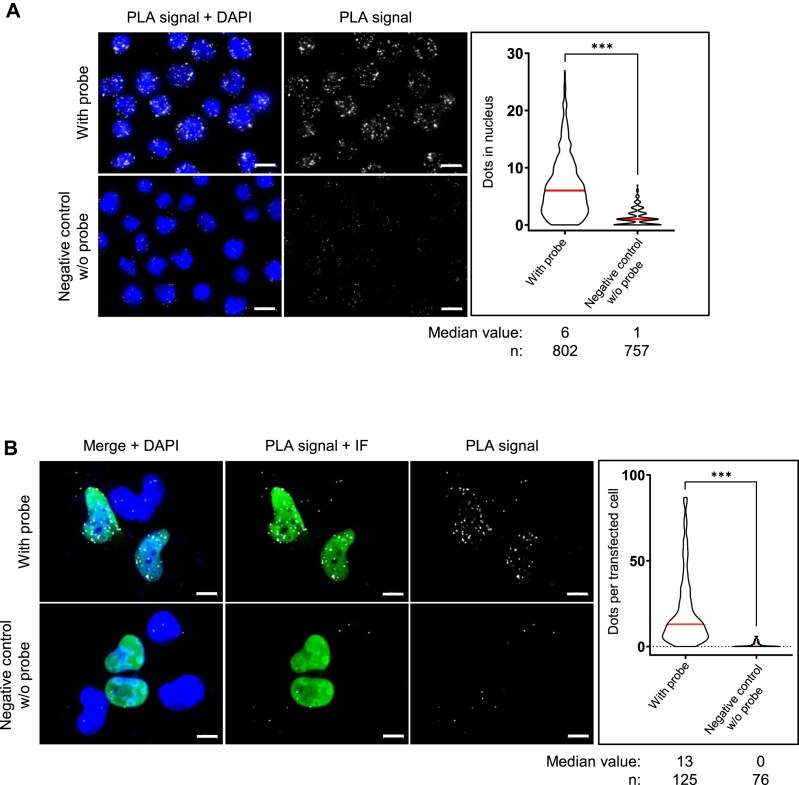
EBNA1 protein*–EBNA1* mRNA interaction mostly takes place in the nucleus. (**A**) Adaptation of the proximity ligation assay (PLA) to monitor protein–RNA interaction performed in Raji cells (EBV-infected Burkitt lymphoma cells) endogenously expressing EBNA1. Microscopy images analysed using a probe specifically hybridizing to the *EBNA1* mRNA (upper panels) or not (lower panels, negative control) as indicated. Nuclei were revealed by DAPI staining and appear in blue; white dots (PLA signals) indicate interaction between EBNA1 protein and *EBNA1* mRNA. The graph on the right indicates the number of PLA dots per cell in each condition. Data were analysed by a non-parametric Mann–Whitney’s test using the GraphPad Prism 8 Software (***P< 0.0001). This experiment was replicated independently four times. White scale bar represents 10 μm. (**B**) Same experiment as in (**A**), except that H1299 cells transfected with triple HA-tagged EBNA1 (3HA-EBNA1) expressing plasmid were used instead of Raji cells. Nuclei were revealed by DAPI staining and appear in blue; white dots (PLA signals) indicate interaction between EBNA1 protein and *EBNA1* mRNA; transfected EBNA1 protein appear in green by immunofluorescence performed with an anti-HA antibody. The graph on the right indicates the number of PLA dots per transfected cells in each condition. Data were analysed by a non-parametric Mann–Whitney’s test using GraphPad Prism 8 Software (***P< 0.0001). This experiment was replicated independently twice. White scale bar represents 10 μm.

To assess whether, and where in the cells endogenously expressed EBNA1 protein interacts with its own mRNA, we used a recently described adaptation of proximity ligation assay (PLA) to monitor protein/RNA interactions [49]. As shown in Fig. 2A, we observed a clear nuclear signal (median value of six dots per cell, most of them being nuclear, as compared to one dot per cell as a median value for the negative control in which the dots were in addition less intense). This confirms, *in cellulo* and with both partners expressed at their physiological level, the interaction between EBNA1 protein and its own mRNA.

We also obtained the same result in another cell type using a 3HA-tagged version of EBNA1 (3HA-EBNA1) transfected in H1299 cells which are derived from metastatic lymph node from lung carcinoma, a cancer which is EBV-independent. For this purpose, we have adapted the previously shown PLA to assess the interaction between ectopically expressed 3HA-EBNA1 and its own mRNA using immunofluorescence (IF) as a proxy to detect transfected cells [25]. As shown in Fig. 2B, numerous PLA dots were observed mostly in the nuclei of cells transfected by the 3HA-EBNA1-expressing plasmid with a median value of thirteen nuclear dots per transfected cells as compared to zero nuclear dot for the negative control without DNA probe.

We concluded that EBNA1 protein and *EBNA1* mRNA indeed interact *in cellulo* and that this interaction mostly takes place in the nucleus, which is in line with the mostly nuclear localization of EBNA1 protein [50–53] and *EBNA1* mRNA [54].

### The binding of EBNA1 protein on rG4 of its own mRNA mainly depends on its two RGG motifs

**Figure 3.**
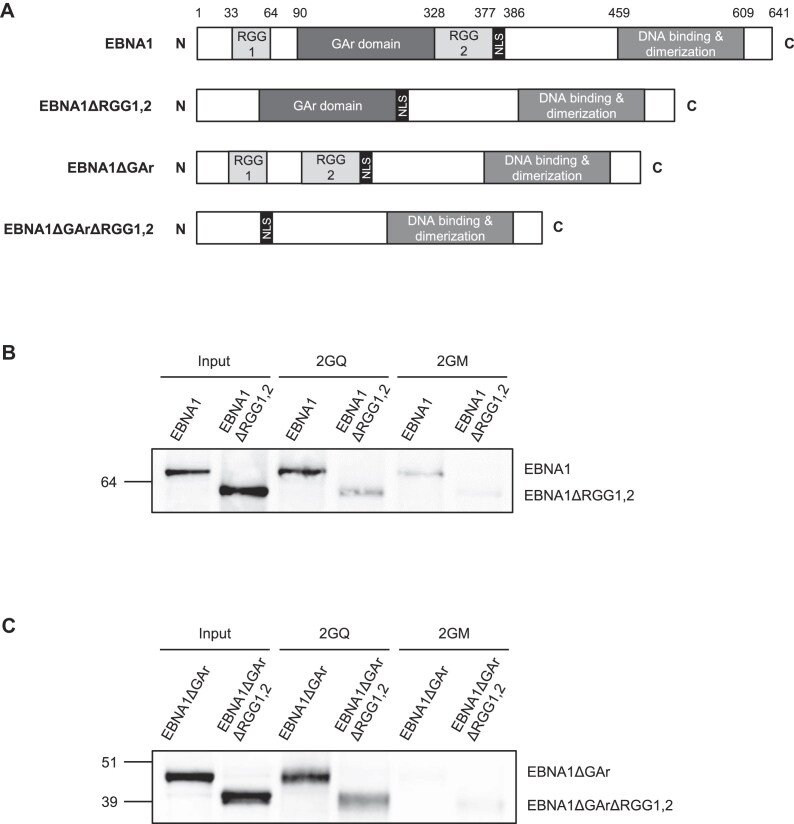
The binding of EBNA1 protein on rG4 of its own mRNA depends mainly on its two RGG motifs. (**A**) Schematic representation of the various constructions used to determine the domain(s) of the EBNA1 protein involved in its ability to bind to rG4 of *EBNA1* mRNA. The numbers above refer to amino-acid positions. NLS: nuclear localization sequence. (**B**) RNA pulldown showing the implication of RGG motifs of EBNA1 to bind to rG4 of its own mRNA. Same RNA pulldown experiments as in Fig. 1A using the 2GQ or 2GM matrices, except that H1299 cells transfected with one or the other of the plasmids expressing the various forms of EBNA1 (EBNA1 or EBNA1ΔRGG1,2) were used instead of Raji cells. The protein still bound after an 800 mM KCl wash were eluted and analysed by SDS-PAGE and western blot using an antibody directed against EBNA1. Blot represents *n* ≥ 3. (**C**) Same experiment as in (**B**) except that plasmids expressing EBNA1ΔGAr or EBNA1ΔGArΔRGG1,2 were used. Blot represents *n* ≥ 3.

As shown in Fig. 3A, EBNA1 contains two RGG motifs: the first one (RGG1) is localized in its *N*-terminal part, upstream of its GAr domain whose mRNA encoding-sequence may form an rG4 cluster, whereas the second one (RGG2) is localized immediately after its GAr domain. To determine if these two RGG motifs of EBNA1 protein may be involved in its ability to interact with rG4 of its own mRNA, we constructed versions of either EBNA1wt (EBNA1) or EBNA1 deleted of its GAr domain (EBNA1ΔGAr) to remove the cluster of rG4 and also to increase the expression of EBNA1, deleted, or not, of these two RGG motifs (respectively termed EBNA1ΔRGG1,2 and EBNA1ΔGArΔRGG1,2) and expressed them in H1299 cells. Using extracts from these cells, we performed RNA pulldown similar to those presented in Fig. 1. As shown in Fig. 3B, the deletion of both RGG1 & 2, despite the fact the corresponding EBNA1 protein (EBNA1ΔRGG1,2) is more expressed, abolishes most of the ability of EBNA1 to interact with rG4 of its own mRNA, with the residual binding being barely superior to the one observed on the 2GM control matrix which cannot form rG4. Of note, the RGG2 motif appears more important than the RGG1 motif for this interaction (Supplementary Fig. 2). We then repeated the same experiment using the versions of EBNA1ΔGAr deleted, or not, for both RGG1 & 2. As shown in Fig. 3C, EBNA1ΔGAr retains its ability to interact with rG4 of *EBNA1* mRNA, and this interaction is G4-dependent as previously observed for EBNA1. In addition, we observed that, as for EBNA1, deletion of RGG1 & 2 significantly affects the ability of EBNA1ΔGAr to bind to rG4 of *EBNA1* mRNA.

Altogether, these results indicate that the ability of EBNA1 protein to interact with rG4 of its own mRNA mainly depends on its two RGG motifs and does not require its GAr domain.

### EBNA1 protein is able to limit its expression and this ability depends on its two RGG motifs

**Figure 4.**
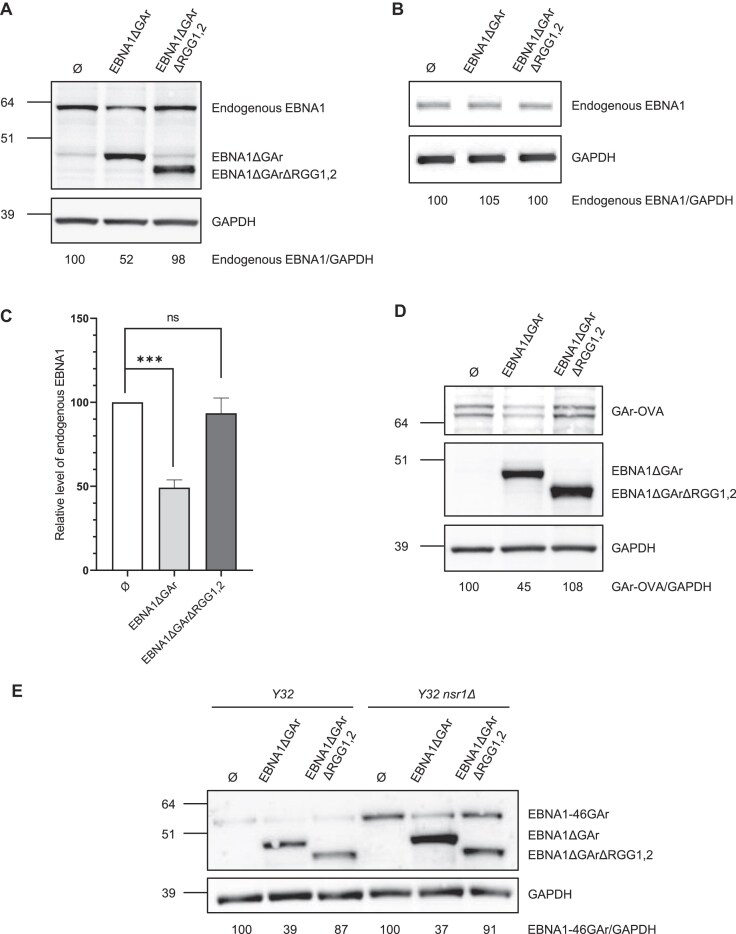
EBNA1 protein is able to self-limit its expression via its interaction with the rG4 of its own mRNA in an RGG-dependent manner. (**A**) SDS-PAGE and western blot analysis of the level of endogenous EBNA1 protein in Raji cells (EBV-infected Burkitt lymphoma cells) in response to EBNA1ΔGAr or EBNA1ΔGArΔRGG1,2 overexpression. GAPDH was used as a loading control. Endogenous EBNA1/GAPDH ratios are indicated below the gel. Blot represents *n*= 4. (**B**) Semi-quantitative RT-PCR experiment to determine the level of endogenous *EBNA1* mRNA from the same Raji cells used in (**A**) overexpressing EBNA1ΔGAr or EBNA1ΔGArΔRGG1,2. The PCR products were analysed by electrophoresis in a 1% agarose gel, revealed by an ethidium bromide staining and quantified by densitometry. GAPDH was used as a loading control. Endogenous EBNA1/GAPDH ratios are indicated below the gel (*n* ≥ 3). (**C**) Statistical analysis of the experiment shown in (**A**). Data were analysed by ANOVA in conjunction with Tukey’s test using GraphPad Prism 8 for Windows (GraphPad Software) (***P < 0.0001). Data are presented as the mean ± standard error of the mean (*n*= 4). (**D**) Same experiment as in (**A**), except that H1299 cells transfected with a GAr-OVA (ovalbumin)-expressing plasmid were used instead of Raji cells. (**E**) SDS-PAGE and western blot analysis of EBNA1-46GAr protein expression when co-expressed with EBNA1ΔGAr or EBNA1ΔGArΔRGG1,2 in an *NSR1wt* yeast strain (*Y32*) as compared to a strain deleted for the *NSR1* gene, the yeast nucleolin homologue (*Y32 nsr1Δ*). GAPDH was used as a loading control. EBNA1-46GAr/GAPDH ratios are indicated below the gel. Blot represents *n* ≥ 3.

We next tested if binding of the EBNA1 protein to the rG4 of its own mRNA could impact, or not, its expression. To address this question, we have adapted a test previously used to demonstrate that NCL regulates EBNA1 expression [20, 38]. Briefly, this test is based on Raji cells which endogenously express EBNA1 as they are chronically infected by EBV. We transfected either EBNA1ΔGAr- or EBNA1ΔGArΔRGG1,2-expressing plasmids in these cells and determined the effect of the resulting overexpression of these two forms of EBNA1ΔGAr on the expression of endogenous EBNA1. We have chosen EBNA1ΔGAr for two reasons: first, because its mRNA does not contain the cluster of rG4 present in *EBNA1* mRNA, hence the resulting overexpressed protein should preferentially interact with mRNA expressed from the endogenous *EBNA1* gene (given that most rG4 of EBNA1 mRNA are clustered within the GAr-encoding sequence); and second, because it is expressed at a higher level than EBNA1 (due to the crucial role of GAr in limiting EBNA1 expression [16, 20]), which is important given that, as non-adherent cells, Raji cells are difficult to transfect. As a negative control, Raji cells were transfected by an empty vector. As shown in Fig. 4A and C, EBNA1ΔGAr overexpression led to a significant decrease (about 50%) of endogenous EBNA1 expression as compared to Raji cells transfected by the empty vector. This effect depends on the RGG motifs of EBNA1ΔGAr, as EBNA1ΔGArΔRGG1,2 had no effect, despite being more expressed than EBNA1ΔGAr. Using semi-quantitative RT-PCR we checked that overexpression of EBNA1ΔGAr or EBNA1ΔGArΔRGG1,2 had no effect on the level of endogenously expressed *EBNA1* RNA (Fig. 4B).

To confirm this effect, we determined the effect of overexpressing EBNA1ΔGAr or EBNA1ΔGArΔRGG1,2 on a GAr-OVA fusion that has been previously demonstrated to be submitted to the same NCL-dependent inhibition of translation than EBNA1 [20, 38]. As shown in Fig. 4D, EBNA1ΔGAr overexpression led to a significant decrease (about 55%) of GAr-OVA expression as compared to H1299 cells transfected by the empty vector. This effect depends on the RGG motifs of EBNA1ΔGAr as EBNA1ΔGArΔRGG1,2 had no effect, despite being more expressed than EBNA1ΔGAr. This result also demonstrates that the interaction between the RGG motifs of EBNA1 protein and its own mRNA depends on rG4s that form in the GAr-encoding sequence of its mRNA.

Finally, to determine if the ability of EBNA1 protein to regulate its own expression could occur in the absence of nucleolin, we tested the effect of overexpressing EBNA1ΔGAr or EBNA1ΔGArΔRGG1,2 in the budding yeast *Saccharomyces cerevisiae*. Indeed, GAr-based inhibition of translation is operant in yeast [32, 55] which has allowed us to setup a yeast-based model to identify drugs able to interfere with this process [32]. This yeast-based system has also allowed us to perform a genetic screening that led to the identification of nucleolin as the first host cell factor critically involved in GAr-based inhibition of translation [20, 30] thanks to the ability of yeast nucleolin (Nsr1p) to bind to rG4 of *EBNA1* mRNA and therefore to limit its translation. Importantly, contrary to NCL in human cells, Nsr1p is not an essential gene in yeast as yeast *nsr1Δ* strains, although growing poorly, are viable [20, 51]. Hence, the yeast system we have developed allows to assess the effect of overexpressing EBNA1ΔGAr or EBNA1ΔGArΔRGG1,2 on EBNA1-46GAr, a form of EBNA1 with a short GAr domain which is operant in yeast, in the presence (*Y32* strain) or absence of nucleolin (*Y32 nsr1Δ* strain). As shown in Fig. 4E, the deletion of *NSR1*, as expected, leads to an increase in EBNA1-46GAr expression. However, we also observed that, irrespectively of the presence or absence of yeast nucleolin Nsr1p, EBNA1ΔGAr, but not EBNA1ΔGArΔRGG1,2, decreases EBNA1-46GAr expression. We concluded that the effect of EBNA1 protein on its own expression may occur in the absence of nucleolin. In addition, the yeast system gives another confirmation of the ability of EBNA1 protein to limit its own expression.

### The interaction between EBNA1 protein and rG4 of its own mRNA may take place in the cytoplasm but needs to be nuclear to impact EBNA1 expression

**Figure 5.**
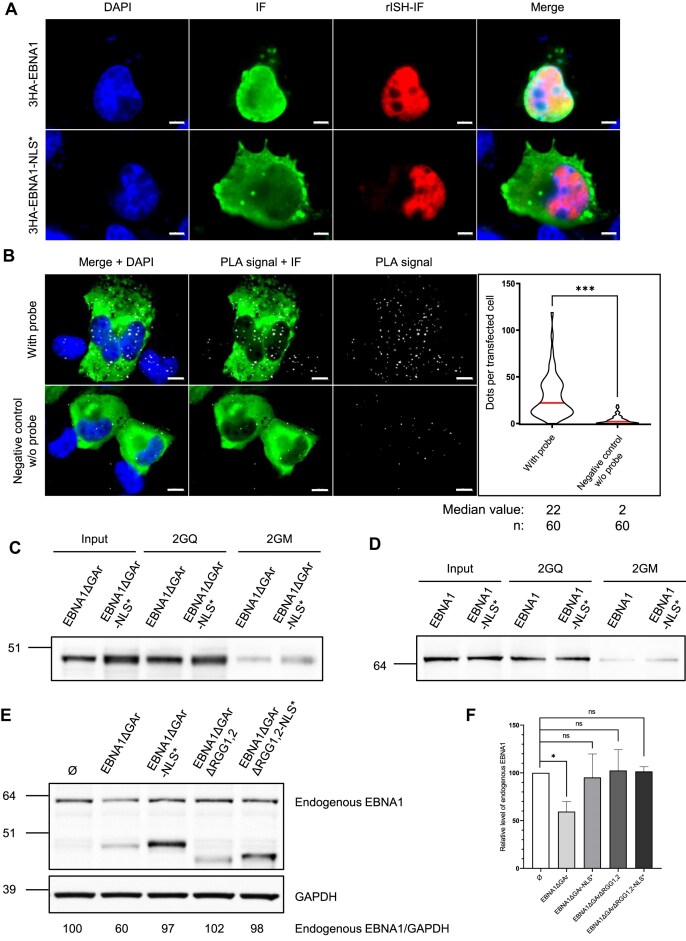
The subcellular localization of EBNA1 protein does not affect its interaction with its own mRNA but impacts the inhibition of its expression. (**A**) H1299 cells expressing the indicated constructs were analysed by RNA *in situ* hybridization - Immunofluorescence (rISH-IF) using probes against *EBNA1* mRNA. Mutation of EBNA1 NLS translocates EBNA1 protein to the cytoplasm, but does not affect the localization of its mRNA. Blue, green, and red represent DAPI, proteins and mRNAs, respectively. White scale bar represents 5 μm. (**B**) Same proximity ligation assay (PLA) experiment as in Fig. 2B performed in H1299 cells transiently expressing 3HA-EBNA1 NLS*. Nuclei were revealed by DAPI staining and appear in blue; white dots (PLA signals) indicate interaction between EBNA1-NLS* protein and EBNA1-NLS* mRNA, green signal in immunofluorescence performed with anti-HA-Tag antibody reveal 3HA-EBNA1-NLS* protein. The graph on the right indicates the number of PLA dots per transfected cells in each condition. Data were analysed by a non-parametric Mann–Whitney’s test using GraphPad Prism 8 Software (***P< 0.0001). This experiment was replicated independently twice. White scale bar represents 10 μm. (**C**) RNA pulldown showing the interaction between EBNA1ΔGAr or EBNA1ΔGAr-NLS* with the rG4 of *EBNA1* mRNA. Same RNA pulldown experiments as in Fig. 3B using the 2GQ or 2GM matrices, except that H1299 cells transfected with EBNA1ΔGAr and also with their NLS mutated version were used. The protein still bound after an 800 mM KCl wash were eluted and analysed by SDS-PAGE and western blot using an antibody directed against EBNA1. Blot represents *n* ≥ 2. (**D**) Same experiments as in (**C**) except that EBNA1 or EBNA1-NLS* were used, as indicated. GAPDH was used as a loading control. Blot represents *n* ≥ 2. (**E**) SDS-PAGE and western blot analysis of the level of endogenous EBNA1 protein in Raji cells (EBV-infected Burkitt lymphoma cells) in response to EBNA1ΔGAr or its NLS-mutated version (EBNA1ΔGAr-NLS*) overexpression. EBNA1ΔGArΔRGG1,2 and its NLS-mutated version were used as negative controls. GAPDH was used as a loading control. Endogenous EBNA1/GAPDH ratios are indicated below the gel. Blot represents *n*= 4. (**F**) Statistical analysis of the experiment shown in (**E**). Data were analysed by ANOVA in conjunction with Tukey’s test using GraphPad Prism 5 for Windows (GraphPad Software) (*P < 0.05). Data are presented as the mean ± standard error of the mean (*n*= 4).

We next determined if the binding of EBNA1 to rG4 of its own mRNA, which mainly takes place in the nucleus, could also take place in the cytoplasm. For this purpose, we have designed an 3HA-tagged version of EBNA1 in which the nuclear localization sequence (NLS) has been mutated (3HA-EBNA1-NLS*). As a control we used a 3HA-tagged version of EBNA1wt (3HA-EBNA1). Each of these two constructs was transfected in H1299 cells in which they represent the sole source of EBNA1, and the localization of both the EBNA1 protein and *EBNA1* RNA was determined using respectively immunofluorescence (IF) and RNA *in situ* hybridization immunofluorescence (rISH-IF). As previously reported [50, 52], and in contrast to EBNA1 protein which is mainly localized in the nucleus, the 3HA-EBNA1-NLS* protein is mainly localized in the cytoplasm as shown in Fig. 5A. Importantly, the localization of 3HA-EBNA1-NLS* RNA remains mainly nuclear, similarly to 3HA-EBNA1. From this result, we concluded that EBNA1 protein is not the main determinant of the nuclear localization of *EBNA1* mRNA.

Next, we performed the same PLA experiment as presented in Fig. 2B to determine if and where 3HA-EBNA1-NLS* protein interacts with its own mRNA. As shown in Fig. 5B, we observed PLA dots both in the cytoplasm and in the nucleus (median value of 22 dots per transfected cells as compared to two nuclear dots per transfected cells of the negative control without probe). We conclude from this result that EBNA1 protein may interact with *EBNA1* mRNA both in the nucleus and in the cytoplasm.

We then sought to determine the impact of the NLS mutation on the ability of EBNA1 protein to interact with rG4 of its own mRNA. For this purpose, we performed RNA pulldown experiments similar to those presented in Fig. 3B and C. As shown in Fig. 5C, EBNA1ΔGAr-NLS* binds to the 2GQ matrix as efficiently as EBNA1ΔGAr, and this binding is mainly rG4-dependent as only a residual binding was observed on the control 2GM matrix. The same results were obtained using extracts of cells expressing EBNA1 or EBNA1-NLS* (Fig. 5D), confirming that this interaction does not depend on the NLS of EBNA1.

Finally, we also determined the impact of the NLS mutation on the ability of EBNA1 protein to limit its own expression. We used the same test based on Raji cells as shown in Fig. 4A and observed that, although being consistently more expressed than EBNA1ΔGAr, and similarly to EBNA1ΔGArΔRGG1,2, EBNA1ΔGAr-NLS* was not able to significantly decrease the steady state level of endogenous EBNA1 protein (Fig. 5E and F). We have also constructed a version of EBNA1ΔGAr that is deleted for its two RGG motifs and that contains a mutated NLS (EBNA1ΔGArΔRGG1,2-NLS*). Again, although being more expressed than the EBNA1ΔGAr control, this version of EBNA1 had no impact on endogenous EBNA1 expression (Fig. 5E and F).

We concluded that, although both cytoplasmic and nuclear pools of EBNA1 protein are able to interact with *EBNA1* mRNA, the interaction needs to take place in the nucleus in order to impact on EBNA1 expression. This may be related to localization of NCL which is mostly nuclear [56, 57] and may thus be the main determinant of the nuclear localization of *EBNA1* mRNA.

### The ability of EBNA1 protein to limit its own expression depends on arginine methylation of its two RGG motifs

**Figure 6.**
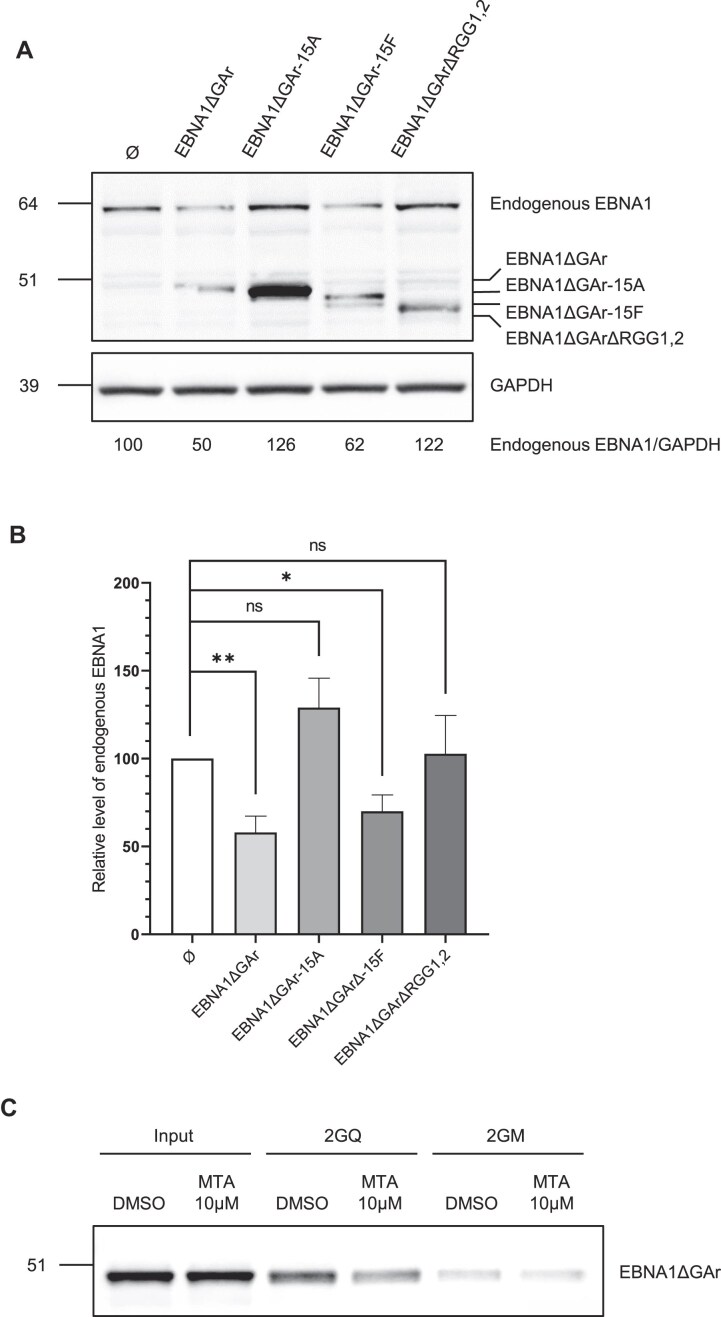
Arginine methylation of the EBNA1 RGG1,2 motifs is important for inhibition of EBNA1 expression and its interaction with rG4 of its own mRNA. (**A**) Same experiment as in Figs 4A and 5E, except that Raji cells were transfected not only with EBNA1ΔGAr but also with its mutated versions (EBNA1ΔGAr-15A or EBNA1ΔGAr-15F) as indicated. Empty vector and EBNA1ΔGArΔRGG1,2 were also used as negative control. GAPDH was used as a loading control. Endogenous EBNA1/GAPDH ratios are indicated below the gel. Blot represents *n*= 4. (**B**) Statistical analysis of the experiment shown in (**A**). Data were analysed by ANOVA in conjunction with Tukey’s test using GraphPad Prism 5 for Windows (GraphPad Software) (*P < 0.05; **P < 0.005). Data are presented as the mean ± standard error of the mean (*n* = 4). (**C**) Same RNA pulldown experiment as in Fig. 3C using extracts from H1299 cells expressing EBNA1ΔGAr except that cells were treated, or not (DMSO control), with 10 μM MTA. The protein still bound after an 800 mM KCl wash were eluted and analysed by SDS-PAGE and western blot by using an antibody directed against EBNA1. Blot represents *n* ≥ 3.

As the ability of NCL to interact with rG4 of *EBNA1* mRNA, and thereby to limit the translation of the latter, depends on the methylation of the arginines of its *C*-terminal RGG motif [38], we next determined if methylation of the arginines of the two RGG motifs of EBNA1 could impact the ability of EBNA1 protein to interact with rG4 of its own mRNA and to inhibit its own expression. For this purpose, we first performed the same experiment as described in Fig. 4A using forms of EBNA1ΔGAr in which the 15 arginines of both RGG1 & 2 have been replaced by either alanines (EBNA1ΔGAr-15A) to prevent methylation, or by phenylalanines (EBNA1ΔGAr-15F), to mimic methylated arginines [38, 58–65]. We used EBNA1ΔGAr as a positive control and EBNA1ΔGArΔRGG1,2 and the empty vector as negative controls. All these vectors have been transfected by electroporation in Raji cells in order to determine their impact on endogenous EBNA1 expression. As shown in Fig. 6A and B, similarly to the two negative controls, EBNA1ΔGAr-15A, although being the more expressed form of EBNA1ΔGAr, had no impact on endogenous EBNA1 expression. In contrast, EBNA1ΔGAr-15F had a significant effect which is close to the one observed for the positive control EBNA1ΔGAr. We concluded that, as for NCL, the methylation of the arginines of the two RGG motifs of EBNA1 protein is important for the ability of the latter to limit its own expression.

An intriguing possibility would be that methylation of the arginines of the two RGG motifs of EBNA1 protein favours the ability of the latter to interact with rG4 of *EBNA1* mRNA. To test this possibility, we performed RNA pulldown experiments similar to the one presented in Fig. 3C using protein extracts form H1299 cells transfected by a construct allowing expression of EBNA1ΔGAr and treated with 5′-methylthioadenosine (MTA), a natural metabolite that is considered to be a general inhibitor of the three types of PRMTs [66] or, as a control, with DMSO (vehicle). As shown in Fig. 6C, MTA leads to a significant, but not total, decrease in the ability of EBNA1ΔGAr to interact with rG4 of *EBNA1* mRNA, indicating that PRMTs indeed control the interaction of EBNA1 protein with rG4 of its own mRNA. Of note, the fact that EBNA1ΔGAr was still able (although significantly less) to interact with rG4 of EBNA1 mRNA suggests that the effect of arginine methylation may indirectly impact the interaction between rG4 of *EBNA1* mRNA and RGG1 & 2 motifs of EBNA1 protein.

### EBNA1 and NCL interact with each other and this interaction favours the binding of both proteins to rG4 of *EBNA1* mRNA

**Figure 7.**
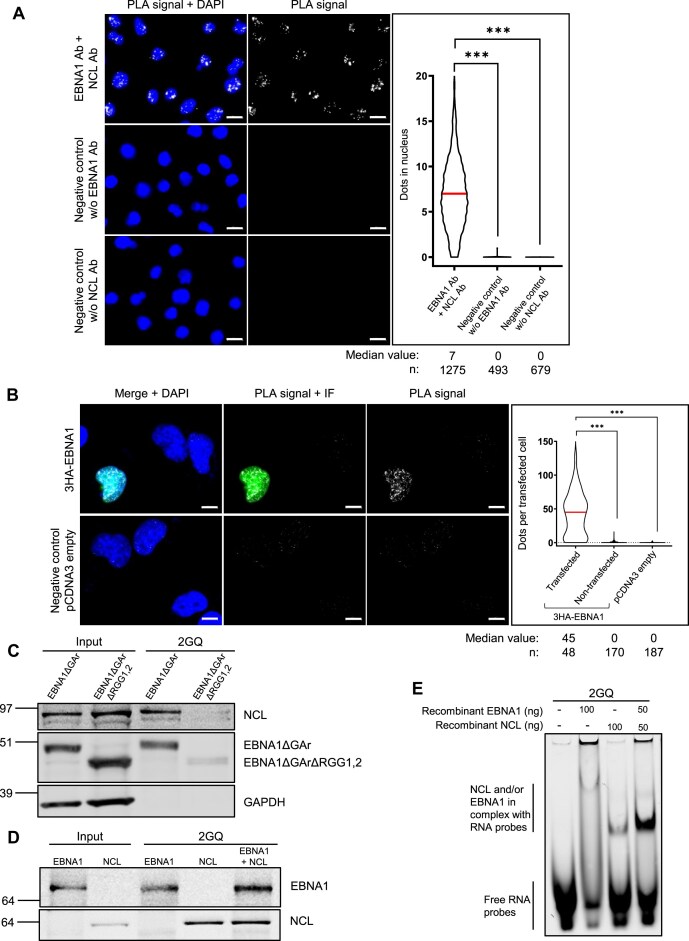
EBNA1 protein and NCL protein cooperate for their binding on rG4 of *EBNA1* mRNA. (**A**) Proximity ligation assay (PLA) for monitoring protein/protein interaction performed in Raji cells natively expressing endogenous EBNA1. Nuclei were revealed by DAPI staining and appear in blue; white dots (PLA signals) indicate interaction between EBNA1 and NCL proteins. The graph on the right indicates the number of PLA dots per nucleus in each condition. Data were analysed by a non-parametric Mann–Whitney’s test using GraphPad Prism 8 Software (*** *P*< 0.0001). This experiment was replicated independently three times. White scale bar represents 10 μm. (**B**) Same PLA experiment as in (**A**), except that H1299 cells transfected with triple HA-tagged EBNA1 (3HA-EBNA1)-expressing plasmid were used instead of Raji cells. Nuclei were revealed by DAPI staining and appear in blue; white dots (PLA signals) indicate interaction between EBNA1 and NCL protein; exogenously expressed EBNA1 protein appear in green in immunofluorescence experiments performed with anti-HA-Tag antibody. The graph on the right indicates the number of PLA dots per nucleus in each condition. Data were analysed by a non-parametric Mann-Whitney's test using GraphPad Prism 8 Software (***P< 0.0001). This experiment was replicated independently twice. White scale bar represents 10 μm. (**C**) Same RNA pulldown experiment as in Fig. 3C performed with cell extracts from H1299 cells transfected with plasmids allowing expression of EBNA1ΔGAr or EBNA1ΔGArΔRGG1,2 as indicated, except that the western blot was revealed by antibodies directed against either NCL (upper panel), EBNA1 (middle panel) or GAPDH (lower panel). (**D**) Same RNA pulldown as in Fig. 1C, except that either recombinant EBNA1, or recombinant NCL, or both, were used. Blot represents *n* ≥ 3. (**E**) EMSA analysis of the binding of either recombinant NCL (100 ng) or recombinant EBNA1 (100 ng) proteins alone, or together (50 ng each) to Cy5.5′-labelled 2GQ (containing two rG4 of *EBNA1* mRNA). Gel represents *n* = 5.

EBNA1 and NCL proteins have been shown to interact with each other, and this interaction depends on the RRM motifs of NCL and on the hundred first amino acids of EBNA1, a region that encompasses the RGG1 motif of EBNA1 [67]. We confirmed this interaction using PLA both in Raji cells that endogenously express EBNA1 (Fig. 7A) and in H1299 cells transfected by a plasmid driving expression of 3HA-tagged EBNA1 (Fig. 7B). In addition, both EBNA1 (this study) and NCL [20] proteins interact directly with rG4 of *EBNA1* mRNA. Hence, we sought to determine if any kind of cooperation may exist between EBNA1 and NCL proteins with respect to their binding to rG4 of *EBNA1* mRNA, or whether the binding of these two proteins is mutually exclusive. For this purpose, we performed the same RNA pulldown experiments as in Fig. 3C using extracts from H1299 cells transfected by plasmids allowing expression of either EBNA1ΔGAr or EBNA1ΔGArΔRGG1,2 proteins and analysed the resulting western blot with anti-EBNA1 or anti-NCL antibodies. As shown in Fig. 7C, and as previously observed (Fig. 3C), the binding of EBNA1ΔGArRGG1,2 protein to the most probable rG4 of *EBNA1* mRNA is much weaker than the one of EBNA1ΔGAr. Importantly, we also observed that the binding of endogenous NCL protein to the same RNA matrix (top panel) is much weaker when EBNA1ΔGArRGG1,2 protein is expressed, as compared to EBNA1ΔGAr, readily suggesting that EBNA1 protein may favour the binding of NCL to rG4 of *EBNA1* mRNA.

In order to directly test this hypothesis, we performed the same RNA pulldown as in Fig. 1 using either recombinant EBNA1, or recombinant NCL, or both. As shown in Fig. 7D, we observed that, in the presence of recombinant EBNA1, the ability of recombinant NCL to bind to an RNA matrix containing two tandem copies of the prototypical rG4 of *EBNA1* mRNA (2GQ) was significantly increased and, reciprocally, the binding of recombinant EBNA1 is increased in the presence of recombinant NCL. The same result was obtained when using an RNA matrix containing only one copy of the prototypical rG4 of *EBNA1* mRNA (1GQ) (Supplementary Fig. 1). We concluded that EBNA1 protein favours NCL binding to rG4 of *EBNA1* mRNA and *vice versa*. This capacity is most probably associated to the ability of EBNA1 and NCL proteins to interact. To confirm these results, we performed electrophoretic mobility shift assay (EMSA) using recombinant EBNA1 and/or NCL proteins, and a fluorescently labelled 2GQ RNA oligonucleotide (Fig. 7E). As expected, by adding independently recombinant EBNA1 (100 ng), or recombinant NCL (100 ng), to the 2GQ RNA matrix, we first observed that each of these two proteins forms a complex with 2GQ RNA nucleotide. This binding is mostly rG4-dependent as only residual binding was observed on a control fluorescently labelled 2GM RNA nucleotide (Supplementary Fig. 3). We then added together half of the quantity (50 ng) of both EBNA1 and NCL proteins and observed a strong increase in the signal, suggesting that the binding of each protein favours the binding of the other on rG4 of *EBNA1* mRNA (Fig. 7E and Supplementary Fig. 3). Finally, by using an increasing quantity (0–40 ng) of recombinant EBNA1, we observed that the binding of a fixed quantity (50 ng) of NCL to rG4 of EBNA1 mRNA was increased in a dose-dependent manner (Supplementary Fig. 4), confirming that EBNA1 favours the interaction between NCL and rG4 of *EBNA1* mRNA.

### The interaction between EBNA1 and NCL proteins is controlled by type I PRMTs and rG4

**Figure 8.**
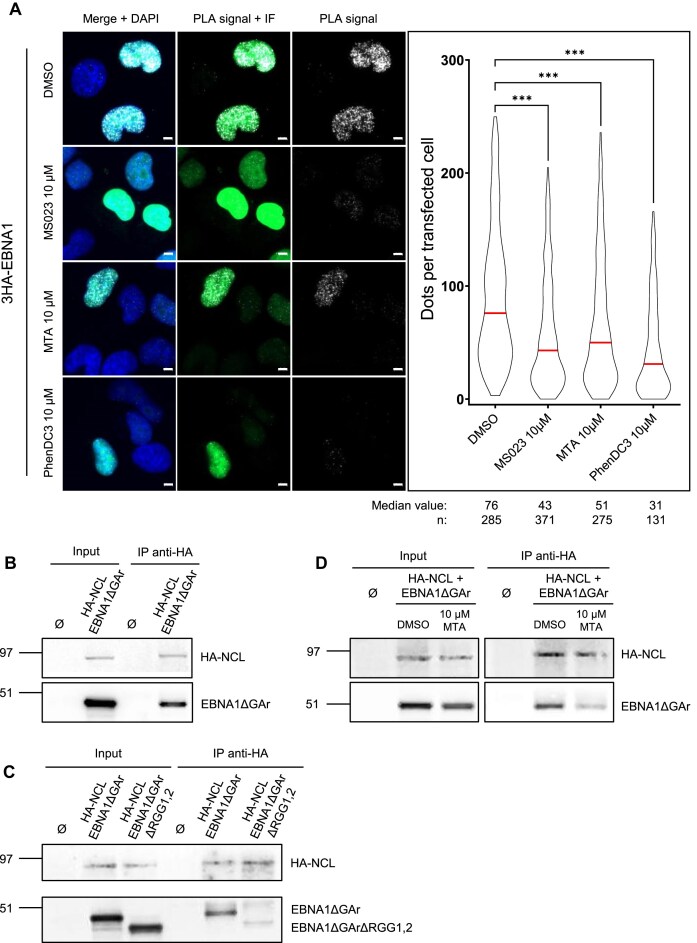
MS023, an inhibitor of type I PRMTs, or PhenDC3, a G4 ligand, decrease the interaction between EBNA1 and NCL proteins. (**A**) Same PLA experiments as in Fig. 7B except that H1299 cells transfected with triple HA-tagged EBNA1 (3HA-EBNA1)-expressing plasmid were treated with either 10 μM of the type I-PRMT inhibitor MS023, 10 μM of the pan-PRMTs inhibitor MTA, 10 μM of PhenDC3, a benchmark G4 ligand or, as a control, with DMSO (vehicle). The graphs on the right indicate the number of PLA dots per transfected cell in each condition. Data were analysed by a non-parametric Mann–Whitney’s test using GraphPad Prism 8 Software (***P< 0.0001). This experiment was replicated independently three times. White scale bar represents 5 μm. (**B**) Co-immunoprecipitation experiment using extracts from H1299 cells transfected, or not with both HA-tagged NCL (HA-NCL)- and EBNA1ΔGAr-expressing plasmids. An anti-HA antibody was used to immune-precipitate HA-NCL and an anti-EBNA1 antibody was used to detect the presence or absence of EBNA1ΔGAr. (**C**) Same co-immunoprecipitation experiment as in (**B**) except that H1299 cells transfected by both HA-tagged NCL (HA-NCL)- and EBNA1ΔGArΔRGG1,2-expressing plasmids were also used to determine the importance of RGG motifs of EBNA1 in its ability to interact with NCL. (**D**) Same co-immunoprecipitation experiment as in (**B**) except that cells were treated with 10 μM MTA or, as a control only by the vehicle (DMSO).

The interaction between EBNA1 and NCL proteins depends on the hundred first amino acids of EBNA1 which encompass the RGG1 motif of EBNA1, thus leaving the possibility that type I PRMT-mediated arginine methylation of EBNA1 may control this interaction. To test this hypothesis, we performed the same PLA experiments as in Fig. 7B, in presence or absence of 10 μM MS023 (a specific inhibitor of type I PRMTs [68]), or of 10 μM MTA (the pan-PRMTs inhibitor used above). Both MS023 and MTA led to a significant decrease in the number and in the intensity of PLA dots indicating that type I PRMTs control the interaction between EBNA1 and NCL proteins (Fig. 8A, upper panels).

Altogether these results suggest a model in which the interaction between EBNA1 and NCL proteins favours the interaction of each of them with rG4 of *EBNA1* mRNA. In addition, the interactions at the basis of this three-way partnership are controlled by methylation of the arginines of the RGG motifs of NCL and/or EBNA1. To further test this ‘*ménage à trois*’ model, we tested the effect of PhenDC3, a G4-ligand which interferes with the binding of both NCL [20] and EBNA1 (this study) to rG4 of *EBNA1* mRNA, on the interaction between EBNA1 and NCL proteins. In a manner similar to MTA or MS023, we observed that 10 μM PhenDC3 significantly decrease the interaction between EBNA1 and NCL proteins, in good agreement with the formation of a ternary complex between both partners and rG4 of *EBNA1* mRNA. Finally, using co-immunoprecipitation experiments, we first confirmed that HA-NCL does interact with EBNA1ΔGAr (Fig. 8B) and that this interaction depends on the RGG motifs of EBNA1 (Fig. 8C). We then observed that MTA (Fig. 8D) or MS023 (Supplementary Fig. 5) strongly reduces the interaction between HA-NCL and EBNA1ΔGAr proteins, in line with the model proposed above in which the three-way partnership between NCL and EBNA1 proteins on the one hand, and *EBNA1* mRNA on the other, is favoured by methylation of the arginines of the RGG motifs of NCL and/or EBNA1.

## Discussion

In this paper, using RNA pulldown and PLA adapted for monitoring protein/RNA interactions, we first show that EBNA1 protein interacts directly with rG4 of its own mRNA and that this interaction mostly takes place in the nucleus, in line with the localization of both partners which are mostly found in the nucleus. We also show that this interaction involves the two RGG motifs of EBNA1 protein. In addition, we found that this interaction limits EBNA1 expression and that this effect depends on type I PRMT activity and methylation of the arginines of the two RGG motifs of EBNA1. Hence, similarly to NCL, EBNA1 protein binds in an RGG motif-dependent manner to rG4 of *EBNA1* mRNA and interaction of both these proteins with *EBNA1* mRNA limits its expression, allowing immune evasion of EBNA1 and thus of EBV. Interestingly, the effect of both EBNA1 and NCL in limitation of EBNA1 expression depends on type I PRMTs further confirming that these enzymes represent a relevant intervention point to interfere with immune evasion of EBV [19, 38]. Finally, our data also suggest the formation of a ternary complex between NCL and EBNA1 proteins and *EBNA1* mRNA, thus leading to the intriguing possibility that both EBNA1 and NCL proteins cooperate to bind to and/or to limit the translation of *EBNA1* mRNA. Importantly, this mechanism, by giving a role to EBNA1 protein in limitation of its own expression, has two important consequences. Firstly, it reconciliates the two main hypotheses to explain how EBNA1 may control its own expression in order to evade the immune system: one giving a role only to *EBNA1* mRNA, in particular to its rG4-forming GAr-encoding sequence, and one giving a role only to EBNA1 protein. Indeed, the results presented in this paper clearly indicate a role of both *EBNA1* mRNA and EBNA1 protein in the mechanism allowing immune evasion of the virus. The second important consequence of our results is that, by giving a direct role to EBNA1 protein in regulation of its own expression, they open the possibility of an autoregulatory loop in which EBNA1 protein may feedback on limitation of translation of its own mRNA. Of note, this potential autoregulatory loop complements nicely the role of NCL in EBV immune evasion [19, 38] as well as its role in EBV episome maintenance and transcription which also involves a direct interaction between NCL and EBNA1 proteins [67]. Indeed, from the virus point of view, it makes sense to have the same host cell protein regulating these three key aspects of EBV's latency. Indeed, if NCL is weakly expressed in the host of the infection, as a result, the maintenance and transcription of EBV episome will be compromised but, as a direct consequence of the role of NCL in GAr-based limitation of EBNA1 expression, *EBNA1* mRNA will be more efficiently translated, which may compensate for its reduced level, thereby allowing the maintenance of EBV genome. On the contrary, if NCL is strongly expressed, then EBV episome will be efficiently maintained and transcribed, hence leading to a high level of *EBNA1* mRNA, but then, due to the role of NCL in self-limitation of EBNA1 expression, its translation will be further inhibited, thus limiting the level of EBNA1 protein and thereby its detection by the immune system. Hence, the involvement of NCL in limitation of EBNA1 expression as well as in the maintenance and transcription of EBV episome may allow to couple and coordinate the synthesis of *EBNA1* mRNA to its translation. The results reported here add an additional layer of regulation in which the level of EBNA1 protein could also feedback on the system controlling its own expression.

**Figure 9.**
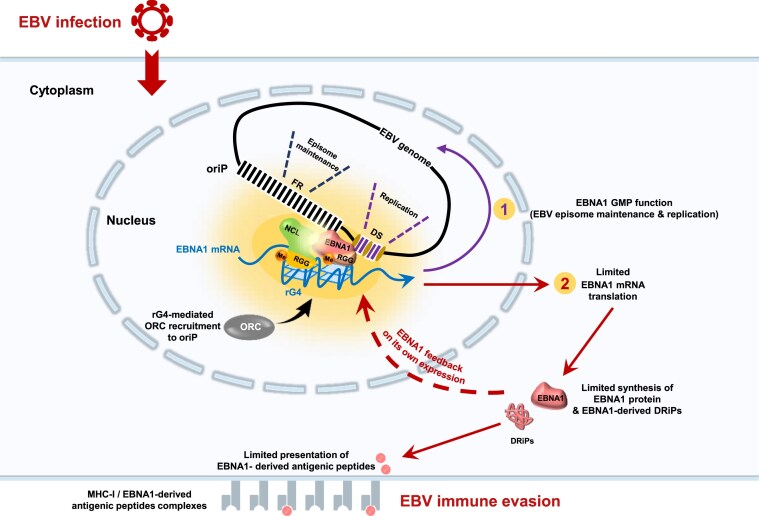
Model of the potential double role of the EBNA1/NCL/*EBNA1* mRNA protein/RNA complex in both the immune evasion and the genome maintenance function of EBNA1. *EBNA1* mRNA is translated in the cytoplasm and nascent EBNA1 protein would directly interact with rG4 of its own mRNA *via* its two RGG motifs. A ternary complex with NCL may form and be imported in the nucleus thanks to the NLS of both EBNA1 and NCL proteins. Within the nucleus, this complex would serve both for the genome maintenance function of EBNA1 (which requires an rG4-dependent interaction with an RNA) and for limiting *EBNA1* mRNA translation, possibly by sequestrating *EBNA1* mRNA in the nucleus. DRIPs: defective ribosomal products.

Another important aspect of the results presented herein and suggesting the existence of a ternary EBNA1/NCL/*EBNA1* mRNA protein/RNA complex is that this complex could serve both to regulate EBNA1 expression to evade the immune system and also for the genome maintenance function of EBNA1. Indeed, the role of EBNA1 in DNA replication and metaphase chromosome attachment, i.e., its viral genome maintenance function, has been shown to be linked to its ability to bind RNA in an rG4-dependent manner [43, 45]. Hence a model in which the same EBNA1/NCL/*EBNA1* mRNA protein/RNA complex is used both for limiting EBNA1 expression below the threshold to be recognized by the immune system and for performing the genome maintenance function of EBNA1 can be envisioned (Fig. 9). In this model, nascent EBNA1 protein could directly interact with its own mRNA and/or in parallel, with NCL, and this ternary complex would be able to both limit EBNA1 expression and perform the genome maintenance function of EBNA1, thereby “killing two birds with one stone” and allowing coordination of these two processes. Of note, as both EBNA1 and NCL are mostly nuclear proteins, in line with the fact they both possess a nuclear localization signal (NLS), altogether they may be responsible for the mostly nuclear localization of *EBNA1* mRNA. For EBV, this nuclear localization of *EBNA1* mRNA may be important for two reasons: firstly, the nucleus is the cellular compartment where the EBNA1/NCL/*EBNA1* mRNA protein/RNA complex may exert its genome maintenance function, hence allowing persistence of the virus, and secondly, this nuclear retention of *EBNA1* mRNA may be responsible, at least in part, for the limited translation of *EBNA1* mRNA, hence limiting the production of EBNA1-derived antigenic peptides which, ultimately, leads to immune evasion of the virus. Of note, in contrast with host genes, most EBV's early and late genes, including *EBNA1*, are intronless (like most viral mRNA, notably of herpesviruses). Given that nuclear export of mRNAs is tightly coupled to transcription, efficient expression of intronless herpesviruses genes requires a specific mechanism for their mRNA export and translation. In case of EBV, nuclear export of intronless viral mRNAs is facilitated by the essential virus-encoded RNA-binding protein EB2 [69–76]. This way, viral mRNAs may be efficiently translated by cytoplasmic ribosomes. However, *EBNA1* mRNA has been reported to be mainly localized in the nucleus ([54, 77, 78] and this study). Although nuclear retention of mature mRNAs is not a common situation, it has been described for a handful of mRNAs (see [79] for a review) and has been proposed to represent an additional mean to regulate gene expression. Hence, a competition between the export and the import or the nuclear retention of *EBNA1* mRNA may exist and thus regulate its translation by cytoplasmic ribosomes.

Another intriguing point is the difference in the relative abundance of NCL and EBNA1 proteins in EBV-infected cells. Indeed, NCL is one of the most abundant nuclear proteins whereas, due to the mechanism self-limiting its own expression, EBNA1 is much less abundant. However, nascent EBNA1 protein is most probably the first in contact with *EBNA1* mRNA in the cytoplasm, a cell compartment in which NCL is scarcely present. Another way to solve this protein level issue may be a differential affinity of both proteins for rG4 of *EBNA1* mRNA.

To conclude, whatever the exact mechanism by which EBNA1 protein participates, together with NCL, to the limitation of EBNA1 expression, it allows the existence of potential auto-regulatory loops. Another important conclusion of the work presented here, and of the resulting model (Fig. 9), is that it highlights two potential therapeutic strategies that may be exploited to reveal EBV-related cancers to the immune system: by targeting either rG4 of *EBNA1* mRNA (for example by means of G4-ligands), or type I PRMTs, and therefore both the interaction between EBNA1 and NCL proteins (this study) and also the interaction between NCL and rG4 of *EBNA1* mRNA [38]. Importantly, proofs of principle for the validity of these two approaches are available ([20, 33, 38] and this study).

## Supplementary Material

gkaf586_Supplemental_File

## Data Availability

The data underlying this article are available in the article and in its online supplementary material.

## Notes

Present address: Laboratoire de Chimie et Biochimie Pharmacologiques et Toxicologiques (LCBPT), CNRS UMR8601, Université Paris Cité, 75006 Paris, France
