# Supplementary material for: The Epstein-Barr virus EBNA1 protein binds to G-quadruplexes in its own mRNA hence controlling its expression and immune evasion of the virus
Source: Nucleic Acids Res. 2025 Jul 4;53(12):gkaf586. doi: 10.1093/nar/gkaf586 (PMC12231556; doi:10.1093/nar/gkaf586)
Supplement: gkaf586_Supplemental_File [file gkaf586_supplemental_file.pdf]

Supplementary figure 1

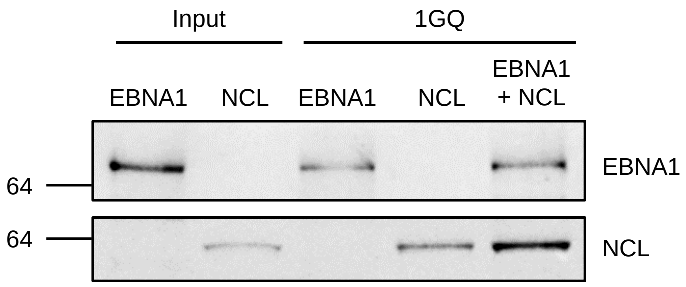

Same RNA pulldown experiments as in **Figure 7d**, except that 1GQ (containing only one rG4 of EBNA1 mRNA) and 1GM (same sequence as 1GQ except that guanines critical for G4 formation were replaced by adenines or uridines) matrices were used. The proteins still bound after an 800 mM KCl wash were eluted and analysed by SDS-PAGE and western blot by using an antibody directed against EBNA1 or NCL. Blot represents $n \geq 3$.

Supplementary figure 2

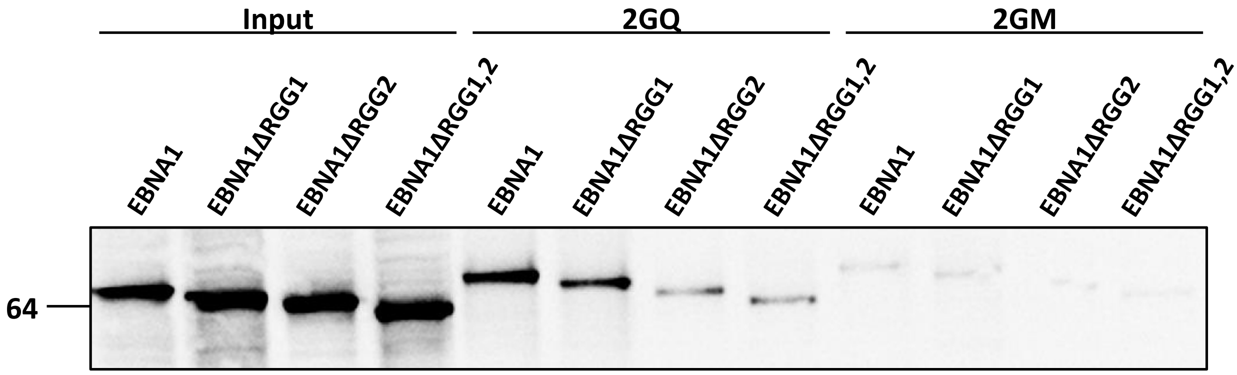

Same RNA pulldown experiments as in **Figure 3b**, except that extracts from H1299 cells expressing EBNA1ΔRGG1 or EBNA1ΔRGG2 (respectively deleted for RGG1 or RGG2 motif of EBNA1) were also used in addition to extracts of H1299 cells expressing EBNA1 or EBNA1ΔRGG1,2 (deleted for both RGG1 & 2 motifs). The proteins still bound after an 800 mM KCl wash were eluted and analysed by SDS-PAGE and western blot by using an antibody directed against EBNA1.

Supplementary figure 3

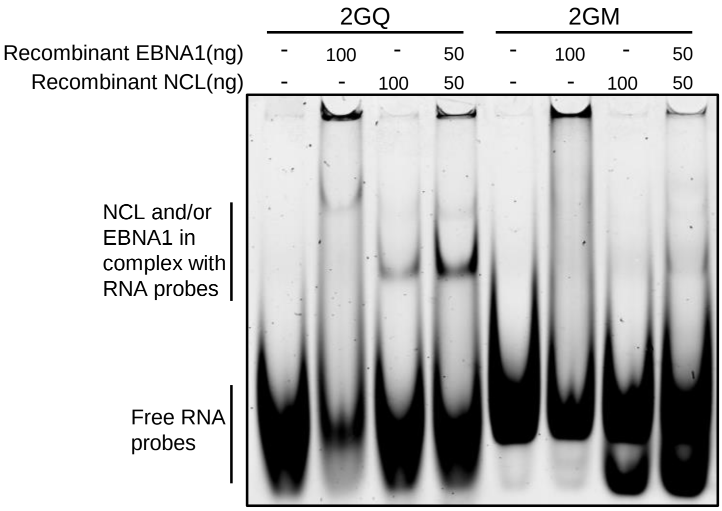

Same EMSA experiment as in **Figure 7e**, using a Cy5.5'-labelled 2GQ RNA probe (containing two rG4 of GAr-encoding sequence of *EBNA1* mRNA) or, as a control, a Cy5.5-labelled 2GM RNA probe (that cannot form rG4). Note that the binding of both EBNA1 and NCL proteins on rG4 of *EBNA1* mRNA is rG4-dependent.

Supplementary figure 4

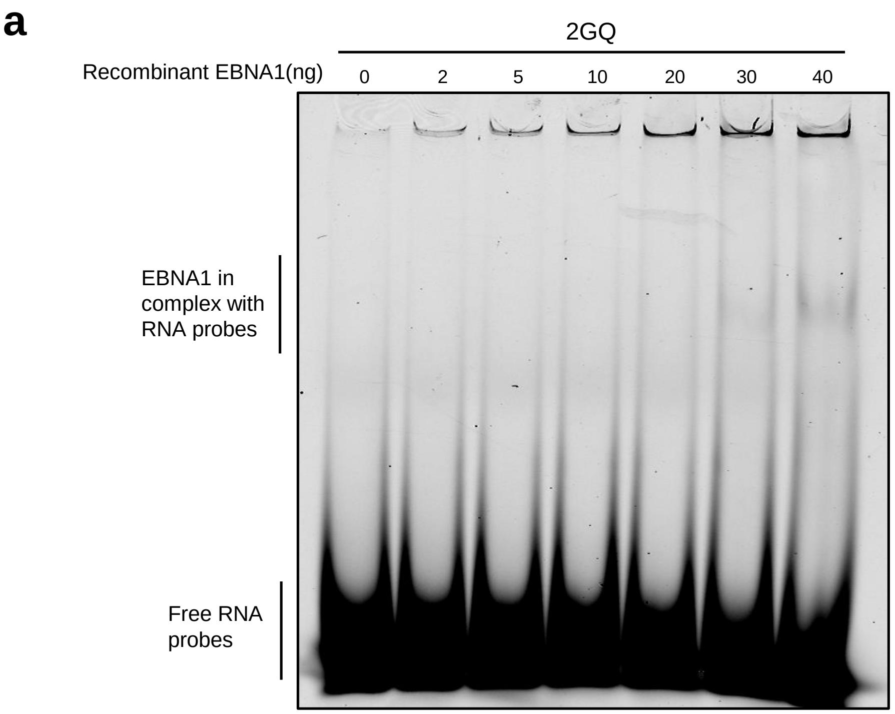

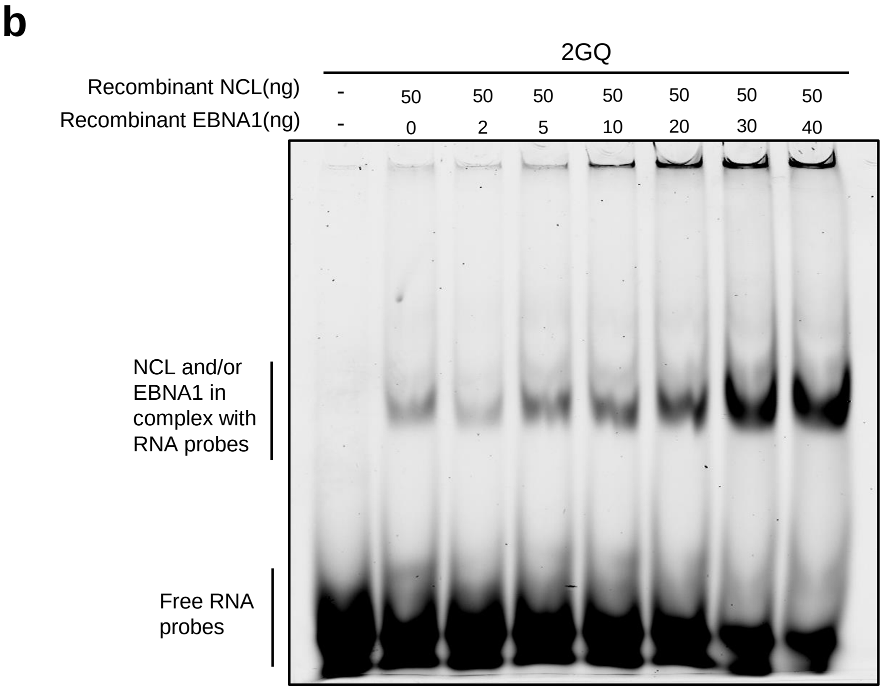

a Same EMSA experiment as in **Figure 7e**, using a Cy5.5'-labelled 2GQ RNA probe (containing two rG4 of GAr-encoding sequence of *EBNA1* mRNA) and increasing quantities of recombinant EBNA1, as indicated. Note that a significant binding of EBNA1 was observed only with 40 ng of EBNA1. **b** Same EMSA experiment using 50 ng of recombinant NCL and increasing quantities of recombinant EBNA1, as indicated. Note that EBNA1 favours, in a dose-dependent manner, the binding of NCL.

Supplementary figure 5

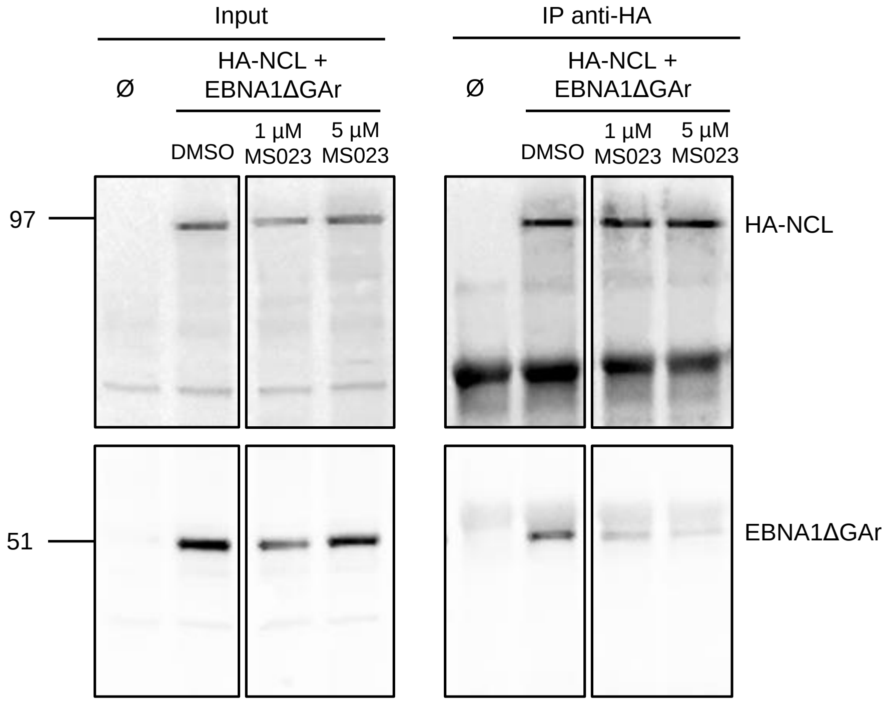

Same co-immunoprecipitation experiment as in **Figure 8d**, except that H1299 cells were treated, or not (DMSO) with MS023 (at 1 µM or 5 µM, as indicated), a specific inhibitor of type I PRMTs.
